# Chicago Medical Society

**Published:** 1885-04

**Authors:** 


At the regular meeting of this Society, held on the evening of February 2, 1885, Dr. Boerne Bettman read a paper entitled "*Peroxide of Hydrogen in Aural and Ophthalmic Practice.*" The essayist remarked that the antiseptic measures introduced by Lister had been applied in aural therapeutics, and marked an era of rational medication in otology. Carbolic acid, although highly satisfactory as a cleansing agent, could not be applied as a germicide, a 5 per cent. solution not being tolerated by the sensitive mucous membrane. The boracic acid treatment of Bezold, therefore, immediately found many advocates; it has well sustained the severe tests to which it has been subjected. Happy results have also been achieved with bichloride of mercury and iodoform. A remedy equally efficient, and very useful in cases where the others have failed, is the peroxide of hydrogen, discovered by Thenard in 1818, and introduced into medical literature by Dr. B. W. Richardson, of London, in 1855. It soon fell into disuse, to be entirely forgotten, but it has lately been reclaimed by the unceasing labors of Dr. Coffin, several Parisian surgeons, and by Dr. W. A. Harlan, of this city. It has been principally employed by dentists in the treatment of alveolar abscesses. But few allusions have been made to it in ophthalmic and aural literature. The usual method of preparing pure peroxide of hydrogen, is by decomposing peroxide of barium with hydrochloric acid. It is a highly prized antiseptic. In a pure state it is a colorless, syrupy liquid, of a slightly bitter taste, and possesses a faint odor of chlorine. Its specific gravity is 1.455. It is a highly unstable compound, decomposing rapidly when exposed to air, and liberating its oxygen. It owes its value as a therapeutic agent to this property. M. Miguel, of the Observatoire de Montsouris, in his comparative table, presenting the relative value of the various antiseptics, places peroxide of hydrogen at the head. The writer then related his observations, made with the microscope, of the action of dioxide of hydrogen upon pus. The pus corpuscles and bacteria are put into lively motion, when a drop of peroxide is allowed to flow under the covering glass. Small gas bubbles, the liberated nascent oxygen, are now evolved. The pus corpuscles gradually lose their spherical shape, shrink, assuming a crescentic form, and are heaped together, a mass of detritus. The bacilli are affected in a similar manner. In a few seconds they are transformed into a dead mass, intermixed with the decomposed pus corpuscles, and surrounded by seething bubbles of gas. The therapeutical action of peroxide of hydrogen has been explained as follows: when it comes in contact with pus, the extra O it contains is liberated so rapidly that the hydrogen and sulphur of the tissues immediately combine, resulting in $H_2\,SO_4$ in small quantity, sufficient to glaze the pus-producing area, thus affording an opportunity for the exuding protoplasmic material to organize into new tissue. The remaining unsatisfied atoms of O quickly distend the pus-sac and force out the contents. He had used the remedy in more than thirty cases of otitis media purulenta, and employs a preparation sold by chemists containing 12 volumes of the gas, as follows: the parts are thoroughly cleansed with warm water, and then dried with absorbent cotton, after which 8 to 12 drops of the remedy are instilled into the ear. Contact with pus and diseased tissues sets free the oxygen visible as bubbles, which, united with the expelled pus, forms a seething, frothy mass. Patients rarely complain of pain. After cleansing the parts, the mucous membrane is seen to have assumed a milky white appearance. The liberated gas enters the most hidden recesses of the middle ear, forcibly dislodging the decomposing material, and destroying bacteria embedded in the tissue meshes. If the perforation is small, the remedy can be injected directly into the middle ear. It had also been successfully employed in the treatment of dacrocystitis, and in one case of chronic trachoma which had run its course, but where there still remained a mucous discharge. Several (5) cases of dacrocystitis yielded only to the peroxide of hydrogen, after having been subjected to the routine medication with astringents, carbolic acid, introduction of Bowman's probe, and gelatine bougies.

In the discussion, Dr. R. Tilley stated that he had seen the peroxide of hydrogen solution used in Paris by Palse as a spray in ovariotomy, and also by Landolt in dacrocystitis, but he saw no manifest advantage from its use that should entitle it to a position of preference over certain well-known and well-tried remedies, among which he mentioned permanganate of potassium solution. In observations with the preparation, it was of considerable importance to adhere to the tendency of the most careful workers of the day, and ascertain to just what factor of the peroxide solution the alleged virtue was attributable. It is a fact that the peroxide of hydrogen solution is rendered more permanent by virtue of an acid; and the acid used is sometimes hydrochloric, and sometimes sulphuric acid is used, the former being more generally selected at the present time. The quantity of acid used is very small; but small as it is, it may have considerable influence. It should always be ascertained by the person using it which of the acids is used to facilitate the solution of the peroxide, as minute quantities of sulphuric acid may have very deleterious effects in certain cases. The best test for the presence of the peroxide in solution is a crystal or two of permanganate of potassium.

Dr. W. W. Allport stated that he had used this remedy in alveolor abscesses and pulpless teeth. On filling a cavity of a tooth with the solution, effervescence occurs and assists the cleansing of the cavity.

Dr. J. S. Marshall has treated burrowing abscesses when, for obvious reasons, he did not wish to make an external opening. By injecting a solution freely into such a cavity, the relief was much more rapid and satisfactory than with other agents he had used.

Dr. G. Newkirk has used it in cases which were not blind abscesses, and obtained satisfaction. He found it difficult to obtain the substance reliable, and to keep it good.

Dr. Bettman concurred with the last speaker that it does not keep well, and that it is of importance to obtain a reliable article. He felt sure that its effect on pus was due to the liberation of oxygen when it comes in contact with pus and its associated products.

*Missed Abortion* was the title of the next paper, by Dr. P. O'Connell, in which he related a case, notes of which cover a period of two years. The patient was twenty-six years old, and had one child, two years old. Three months after her last confinement the catamenia appeared. This function continued to come on regularly for several months, when it ceased. In addition, the other usual symptoms of pregnancy were present. Three months from the commencement of pregnancy the patient fell, striking rather heavily on her hands and knees. A slight sanguineous vaginal discharge, lasting a few hours, followed the fall. After this the abdomen, which had been enlarging, slowly diminished to its normal state, and her health slowly but steadily deteriorated. She had almost constant aching, with occasionally a severe pain, and a sensation of the limb "going to sleep" was felt in the left lower extremity. She could not lie on the left side.

Careful palpation of the hypogastrium gave no evidence of uterine enlargment. The left vaginal region was tender on deep pressure. On vaginal examination, the finger met the uterus fully one inch lower than normal, with the os and cervical canal so patent as readily to admit the index finger. The anterior segment of the cervix was thickened. Upon bimanual examination, the uterus was found to be somewhat enlarged, but not sensitive.

The sound penetrated to a depth of 2½ inches, and to the left. There was no hyperæsthesia of the vagina, although its mucous membrane was congested. The cervix was also congested and bled freely and readily. Ergot and strychnia were prescribed, and soon after the first dose was taken uterine contractions set in, resulting in the expulsion of the mass, which was greatly decomposed and abominably ill-smelling. It had doubtless lain in utero for three months. Upon closely questioning the patient, she said that during August, which was extremely hot part of the time, she frequently felt so cold at night as to require heavy bed coverings to keep warm. That she had frequent chills, and once had what would seem a rigor. These symptoms, with itching, an erratic rash on the skin of the lower abdomen and back, together with malaise and slowly increasing debility, clearly pointed to the absorption of septic matter from the cavity of the uterus. The pain in the left lower extremity was due to the dead weight of the uterus on the branches of sacral plexus of nerves. The specimen was then exhibited, which presented a mummified appearance and consisted of the placenta and two fœti.

*Muriate of Apomorphia in Chronic Bronchial Asthma*, with a report of two cases, is the subject of a paper read by Dr. G. W. Webster.

CASE I. Mr. G——, English, 34 years of age, married, carpenter, has suffered from bronchial asthma for more than one year. He has been a resident of the United States about two years and three months. During the past summer, especially at night, he suffered greatly from this difficulty. When first called to attend him, in December last, he was suffering from asthma and severe attacks of spasmodic coughing spells, and he had been for some days confined to his bed. Gave him remedies to overcome the difficulty, which did not in some respects afford him the least relief.

Apomorphia per os in gr. 1-20 every three hours, was given him. And the following morning the patient stated that he passed a comfortable night. The remedy was then increased to 1-10th gr. ter die, and the second night he slept well and rested comfortably from this time on whilst taking the drug. It was then discontinued; but the attacks recurred, however, with much less severity. The remedy was again renewed and with entire relief. It was then given him for two weeks, during which time he was up and about and able to follow his trade.

It is now four weeks since he ceased taking the drug and there has been no return of the disease.

CASE 2. Mr. B——, aged 58, laborer, has had chronic bronchitis with asthma every winter for twenty years. When called to see him in January, found him suffering greatly from dyspnœa; rales were present over the entire chest, and his face and hands were cyanosed, and he was gasping for breath. Gave apomorphia in 1-20 gr. doses every three hours, and the subsequent day he was considerably relieved and could breathe freely. The remedy was continued a few days longer with continued relief to the patient, since which the writer had not seen him.

D. C. T. Fenn referred to a mismanaged case of "*frost-bite*," and alluded to the ill usage of two of our most efficacious remedies, viz., ice and heat, by their being wrongly employed.

The society then adjourned.

